# The genetic susceptibility linking preterm birth and periodontal disease – a review

**DOI:** 10.1080/07853890.2021.1896890

**Published:** 2021-09-28

**Authors:** Joana Couceiro, Ana Rita Grosso, Pedro V. Baptista, José J. Mendes, Alexandra R. Fernandes, Alexandre Quintas

**Affiliations:** a Laboratório de Patologia Molecular e Bioquímica Forense, Centro de Investigação Interdisciplinar Egas Moniz (CiiEM), Egas Moniz Cooperativa de Ensino Superior, Caparica, Portugal; b UCIBIO, Department of Life Sciences, Faculdade de Ciências e Tecnologia, Universidade NOVA de Lisboa, Caparica, Portugal; c Clinical Research Unity, Centro de Investigação Interdisciplinar Egas Moniz (CiiEM), Egas Moniz Cooperativa de Ensino Superior, Caparica, Portugal; d Laboratório de Ciências Forenses e Psicológicas, Centro de Investigação Interdisciplinar Egas Moniz (CiiEM), Egas Moniz Cooperativa de Ensino Superior, Caparica, Portugal

## Abstract

**Introduction:**

Preterm birth (PTB) is a major clinical and public health challenge, being the main determinant of neonatal mortality and the second most common cause of death in children younger than 5 years old [1]. This condition is also responsible for 75% of neonatal morbidity which often extends to later life, resulting physical, psychological and economic costs [2]. Although some risk factors for PTB have already been described, its aetiology is still uncertain [3]. Several inflammatory diseases have been associated to PTB, including periodontal disease (PD), ranking the 6th position among the most prevalent diseases worldwide [4]. Despite the links that have been proposed, the relationship between PTB and PD is not fully understood. Nevertheless, both conditions were associated with a genetic predisposition and relevant variants were found in genes associated with the inflammatory system. The present study aims to shed light on the inflammatory network underlying PTB and PD occurrence.

**Materials and methods:**

A literature review was conducted in B-on, Pubmed and Science Direct databases using the search terms: “preterm birth”; “periodontal disease”; “genetic variants” and “inflammation”. Only peer reviewed papers in English, published between 2000 and 2019 were included. Studies using animal cell lines, animal experiments and in silico research were excluded. Six genes associated to PTB and PD were found. After, STRING biological database was used to predict protein-protein interaction for each of the genes found previously.

**Results:**

Figure 1 shows the STRING networks for IL1A and MMP9, two of the most common genes which variants are associated both with PTB and PD. Afterwards, a search on the literature for the genes in the 1^st^ sphere of the network was conducted to find any correlation with PTB and PD. In the end 90 variants in 40 different genes were identified associated to these conditions. **Discussion and Conclusions:** This novel bibliography research procedure, combining STRING search for protein-protein interaction with a regular search on scientific literature databases, allow to acquire a comprehensive vision of all inflammatory related genes that have been associated to PTB and PD till this very moment.

**Figure 1.**
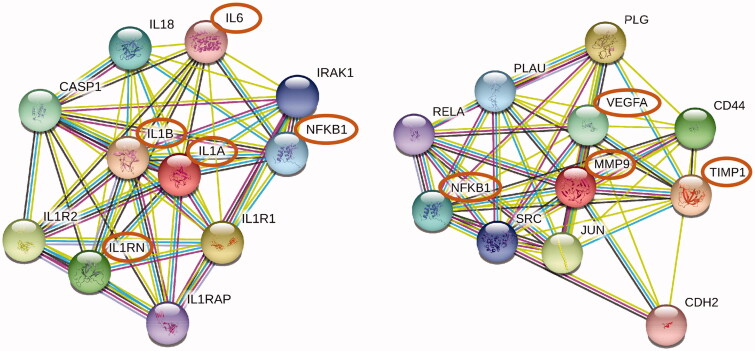
Example of protein networks of two key proteins involved in PTB and PD.
